# Changes in the Homeostatic Appetite System After Weight Loss Reflect a Normalization Toward a Lower Body Weight

**DOI:** 10.1210/clinem/dgaa202

**Published:** 2020-04-17

**Authors:** Julia Nicole DeBenedictis, Siren Nymo, Karoline Haagensli Ollestad, Guro Akersveen Boyesen, Jens Frederik Rehfeld, Jens Juul Holst, Helen Truby, Bard Kulseng, Catia Martins

**Affiliations:** 1 Obesity Research Group, Department of Clinical and Molecular Medicine, Faculty of Medicine, Norwegian University of Science and Technology (NTNU), Trondheim, Norway; 2 Nord-Trøndelag Hospital Trust, Clinic of Surgery, Namsos Hospital, Norway; 3 Department of Clinical Biochemistry, Rigshospitalet, University of Copenhagen, Copenhagen, Denmark; 4 Novo Nordisk Foundation, Center for Basic Metabolic Research and Department of Biomedical Sciences, University of Copenhagen, Copenhagen, Denmark; 5 School of Human Movement and Nutrition Sciences, The University of Queensland, Brisbane, Queensland, Australia; 6 Centre for Obesity and Innovation (ObeCe), Clinic of Surgery, St. Olav University Hospital, Trondheim, Norway

**Keywords:** weight loss, normalization, compensation, ghrelin, hunger

## Abstract

**Objective:**

To compare appetite markers in reduced-obese individuals with a nonobese control group.

**Methods:**

A total of 34 adults with obesity who lost 17% body weight at week 13 and maintained this weight loss (WL) at 1 year were compared with 33 nonobese controls matched for body composition. Basal and postprandial subjective appetite ratings and appetite-related hormone concentrations (ghrelin, total peptide YY, peptide YY_3-36_, total and active glucagon-like peptide 1, and cholecystokinin) were measured in all participants and repeated at week 13 and 1 year in the weight-reduced group.

**Results:**

WL led to a reduction in prospective food consumption and an increase in feelings of hunger, fullness, and ghrelin secretion (basal and postprandial), but these new ratings were no different from those seen in controls. Postprandial concentrations of active glucagon-like peptide 1, total peptide YY, and cholecystokinin were lower in individuals with obesity at all time points compared with controls.

**Conclusion:**

The increased drive to eat (both subjective feelings of hunger and ghrelin concentrations) seen in reduced-obese individuals, both after acute and sustained WL, reflects a normalization toward a lower body weight. Overall, WL does not have a sustained negative impact on satiety peptide secretion, despite a blunted secretion in individuals with obesity compared with nonobese controls.

Obesity is a chronic, progressive, and relapsing condition that is rising in prevalence among the world’s population (1). Lifestyle interventions can lead to clinically relevant weight loss (WL) in the short-term, however, the majority of people experiences weight regain in the long-term (2, 3). To explain for this recidivism, it has been proposed that diet-induced WL triggers compensatory changes in the secretion of appetite-related hormones, which increases hunger and reduces postprandial fullness, leading to overeating and subsequent weight regain (4, 5). Proponents of the theory of “compensation” have cited alterations in homeostatic appetite-hormone secretion in weight-reduced individuals as evidence of this mechanism. For example, WL has been shown to lead to increased secretion of the hunger hormone ghrelin and a reduction in the postprandial secretion of satiety peptides, such as active glucagon-like peptide 1 (GLP-1), total peptide YY (PYY), and cholecystokinin (CCK) (6, 7). However, the weight-regain promoting actions of these “compensatory” changes in appetite remain largely speculative, as evidence which demonstrates a causal relationship between increased appetite after WL and the risk of weight regain is lacking (8).

Sumithran and colleagues (2011) showed in their landmark paper that diet-induced weight loss was associated with a sustained increase in hunger feelings and ghrelin secretion, as well as a reduction in the postprandial release of the satiety hormones total PYY and CCK (6). The authors concluded that strategies to counteract these changes in appetite seen with WL were needed to prevent weight regain. However, the lack of clear associations between changes in homeostatic appetite-regulating hormones during WL and weight regain at 1-year follow-up (6) weakens the argument of this being a compensatory response. We have recently published evidence that the increased hunger feelings and ghrelin secretion, seen with diet-induced WL, are not predictors of long-term weight regain (9). Moreover, studies that have measured different hormonal fractions of satiety peptides (total GLP-1 and PYY_3-36_) have shown an increase in the postprandial release of these hormones with WL, suggesting different methodologies used to measure these labile hormones may be the cause of these inconsistencies in findings (10).

To date, most studies only measure the plasma concentration of appetite-related hormones in individuals with obesity before and after WL, rather than comparing these altered levels of hormonal secretion with stable-weight controls matched to their reduced body weight or fat mass (FM). Verdich et al. (2001), who did use this study design, found that postprandial total GLP-1 secretion after diet-induced WL partially normalized to a level near comparable to a lean control group (11). Moreover, although ghrelin secretion is commonly reported to increase with WL, individuals with obesity present with lower, not higher, basal plasma concentration of ghrelin compared with healthy-weight individuals (7). Individuals with obesity also present with reduced postprandial secretion of GLP-1 and PYY compared with healthy-weight individuals (7).

In light of this evidence, we hypothesized that the changes in the secretion of homoeostatic appetite-regulating hormones after diet-induced WL are not a compensatory response that drives relapse, but result from a normalization of hormone secretion which aligns with the lower body weight and reduction in fat mass. This study tests this hypothesis by determining if the changes in appetite-related hormone secretion after diet-induced WL are similar to or different than those in weight-stable, nonobese controls, matched for body composition (both FM and fat-free mass [FFM]). A secondary aim was to compare subjective feelings of appetite in reduced-obese individuals and these matched nonobese controls.

## Materials and Methods

### Study design

In this study, reduced-obese individuals, who had previously lost 17% of their body weight (through an 8-week very-low-energy diet [VLED] followed by a 4-week refeeding and weight stabilization phase and a 1-year WL maintenance program), were compared with a nonobese FM- and FFM-matched control group. Measurements in reduced-obese individuals were taken at baseline (before WL), at week 13, and 1 year.

### Participants

Data from 34 healthy adults with obesity (body mass index [BMI]: 34.3 ± 2.8 kg/m^2^) who had participated in a previous WL study (12) were used for this analysis. Thirty-three nonobese controls (18.5 < BMI < 29 kg/m^2^) were recruited via online and newspaper advertising in Trondheim, Norway. The study was approved by the regional ethics committee (Ref., 2012/1901) and conducted according to the guidelines laid down in the Declaration of Helsinki. All participants provided written informed consent before commencement.

All participants at recruitment were required to be weight stable (< 2 kg body weight change over the past 3 months), not currently dieting to lose weight, and with a sedentary lifestyle (engaging in < 150 minutes/week of physical activity of at least moderate intensity) (13). Because of the known effect of the phase of the menstrual cycle on appetite, females had to be postmenopausal or taking hormonal contraceptives (14). Exclusion criteria were pregnancy, breastfeeding, and clinically significant illness including diabetes, polycystic ovarian syndrome, hyper-/hypothyroidism, previous WL surgery, and/or medication known to affect appetite/metabolism or induce WL.

Demographic and anthropometric data for both groups can be seen in Table 1. The obese group lost 17% of their baseline weight by week 13 and this WL was maintained at 1 year. A significant reduction in FM (kg, %) and increase in FFM (%) was seen at week 13 and 1 year when compared with baseline (*P* < 0.001 for all), whereas no significant changes were seen for FFM (kg). The 2 groups had a similar sex distribution, but the obese group was significantly older than the nonobese control group (average difference 5 years, *P* = 0.05). Even though the participants after WL presented with a significantly higher body weight and BMI (*P* < 0.001 for both) compared with the nonobese controls, there were no significant differences in body composition (either FM or FFM [% or kg]) between reduced-obese at either Wk13 or 1Y and controls (Table 1)

**Table 1.** General Characteristics of the Participants

	Obese Baseline (n = 34)	Reduced-obese Week 13 (n = 34)	Reduced-obese 1 Year (n = 34)	Controls (n = 33)
Sex (% females)	50%			49%
Age (years)	45.0 ± 1.5^a^			38.9 ± 2.0^a^
Weight (kg)	103.0 ± 2.1^bc^	85.5 ± 2.2^b^	85.7 ± 2.1^c^	76.8 ± 2.1^bc^
BMI (kg/m^2^)	34.0 ± 0.4^bc^	28.2 ± 0.5^b^	28.2 ± 0.4^c^	24.8 ± 0.4^bc^
Fat mass (%)	41.8 ± 1.5^bc^	30.3 ± 1.6^b^	31.5 ± 1.5^c^	29.7 ± 1.5^bc^
Fat mass (kg)	42.9 ± 1.5^bc^	25.7 ± 1.6^b^	27.3 ± 1.5^c^	22.7 ± 1.5^bc^
Fat-free mass (%)	58.2 ± 1.5^bc^	69.7 ± 1.6^b^	68.5 ± 1.5^c^	70.3 ± 1.5^bc^
Fat-free mass (kg)	60.1 ± 1.9	59.5 ± 2.0	58.4 ± 1.9	54.1 ± 1.9

Data presented as estimated marginal means ± SEM. Means with the same superscript letter are significantly different.

BMI, body mass index.

^a^ *P* = 0.05.

^b,c,d^ *P* < 0.001.

### Weight loss and maintenance

The dietary protocol and study design which resulted in the WL has been fully described elsewhere (12). In brief, participants were provided with a VLED for 8 consecutive weeks (Allevo, Karo Pharma AS, Sweden) with an energy intake of 2302/2763 kJ (550/660 kcal) per day for females and males, respectively (macronutrient composition: carbohydrates 42%, protein 36%, fat 18%, and fiber 4%). No-energy fluids were allowed ad libitum. Intake of low-starch vegetables (maximum 100 g/day) were encouraged. At week 9, participants were gradually reintroduced to normal food, whereas reducing intake of the VLED products for 4 weeks aiming at weight stabilization (there was no change in body weight between weeks 9 and 13: -0.3 ± 2.6 kg, *P* = 0.487). An individual diet plan was prescribed by a trained dietitian tailored to individual energy requirements, with 15% to 20% protein, 20% to 30% fat, and 50% to 60% carbohydrates, aimed at weight stabilization. This was followed by a 1-year multidisciplinary weight maintenance program.

### Outcome variables

The following measurements were performed at baseline, week 13, and at 1 year in the WL group and at baseline in nonobese controls.

Body weight and composition was determined by air displacement plethysmography (BodPod, COSMED, Rome, Italy).

#### Appetite measures.

Subjective appetite feelings (hunger, fullness, desire to eat, and prospective food consumption [PFC]) were measured using a validated 10-cm visual analog scale (15) and blood samples were collected in the fasting state and every 30 minutes (0, 30, 60, 90, 120, and 150 minutes) after a standardized breakfast (2512 kJ [600 kcal]: 17% protein, 35% fat, and 48% carbohydrates), for a period of 2.5 hours. Plasma samples were analyzed for active ghrelin, total PYY, and active GLP-1 using a Human Metabolic Hormone Magnetic Bead Panel (LINCOplex Kit, Millipore, St Louis, MO). Total GLP-1 and CCK were measured using “in-house” radioimmunoassay methods (16, 17) and PYY_3-36_ was measured with a commercial radioimmunoassay (Millipore). The intra- and inter-assay coefficients of variation were < 10% and < 15% for ghrelin, active GLP-1, total PYY, and PYY_3-36_; and < 5% and < 15% for total GLP-1 and CCK, respectively. Blood samples were collected in EDTA-coated tubes. One milliliter of whole blood was then transferred into a micro tube and a 20 μL mixture of inhibitors (10 μL of Pefabloc [Roche Diagnostic, Germany] + 10 μL DPP-IV inhibitor [Merck Millipore, Germany]) was added. For CCK, total GLP-1 and PYY_3-36_ analysis, aprotinin (DSM, Coatech AB, Kaiseraugst, Switzerland) (500 KIU/mL whole blood) was added to the EDTA tube. Samples were then centrifuged at 3200 rpm for 10 minutes at 18°C and the plasma frozen at −80°C until further analysis. All the samples from the same participant were analyzed in the same batch. The analysis was performed by the same technician, except for CCK and total GLP-1, which were analyzed at the University of Copenhagen, Denmark.

### Statistical analysis

Statistical analysis was performed with SPSS, version 22 (SPSS Inc., Chicago, IL), and data presented as mean ± SEM, except for baseline anthropometric data, where mean ± SD was used. Statistical significance was set at *P* < 0.05. All data (anthropometrics, subjective appetite feelings, and plasma concentration of appetite-related hormones, both in the fasting state and after the standardized breakfast [as area under the curve—AUC]) were analyzed using linear mixed-effects models with restricted maximum-likelihood estimation. To enable estimation of changes over time in the group with obesity and the 1 observation for the control group within the same model, the model was based on a categorical variable with 4 levels: 1 through 3 for the obese group (baseline, week 13, and 1 year, respectively), and 10 for the control group (baseline). The comparisons of interest were calculated from linear combinations of the estimated means for each of these 4 categories. Within-subject correlations were accounted for by random subject-specific intercepts in the linear mixed-models. Bonferroni correction was used for post hoc pairwise comparisons: nonobese vs obese (t = 1,2,3) + obese (t1-2, t1-3, t2-3); therefore, 6 comparisons. The term “changes over time” refers, therefore, to changes within the obese group (baseline, week 13, and 1 year). AUC for subjective appetite feelings and plasma concentration of appetite-related hormones was calculated from 0 to 150 minutes after the standardized breakfast, using the trapezoid rule and positive incremental AUC (iAUC) presented, as it adjusts for fasting values presented, this was used as it adjusts for fasting values.” Adjusting for menopausal status did not change the study outcomes and, therefore, we decided not include this variable in our linear mixed-effects models.

## Results

No significant changes in the fasting state were seen for subjective feelings of hunger, fullness, desire to eat or PFC over time within the obese group (obese at baseline versus reduced-obese at week 13 and 1 year), nor differences between the obese group, at any time point (baseline, week 13, or 1 year), and weight-stable controls (Table 2). Basal plasma concentration of ghrelin significantly increased with WL compared with baseline (*P* < 0.001 at week 13 and 1 year), but to levels no different than those seen in nonobese controls. However, basal ghrelin plasma concentrations were significantly lower in individuals with obesity at baseline compared with nonobese controls (*P* < 0.001) (Table 2).

**Table 2.** Appetite Variables in the Fasting State

	Obese Baseline (n = 34)	Reduced-obese Week 13 (n = 34)	Reduced-obese 1 Year (n = 34)	Controls (n = 33)
Hunger (cm)	3.6 ± 0.4	4.9 ± 0.4	4.5 ± 0.4	4.4 ± 0.4
Desire to eat (cm)	4.7 ± 0.3	5.0 ± 0.4	4.4 ± 0.3	4.7 ± 0.3
PFC (cm)	6.1 ± 0.4	5.5 ± 0.4	5.3 ± 0.4	6.0 ± 0.4
AG (pmol/mL)	101.8 ± 17.8^abc^	174.3 ± 19.4^a^	167.6 ± 17.8^b^	222.5 ± 18.4^c^

Data presented as estimated marginal means ± SEM. Means with the same superscript letter are significantly different.

AG, acylated ghrelin; PFC, prospective food consumption.

^a,b,c^ *P* < 0.001.

**Figure 1.**
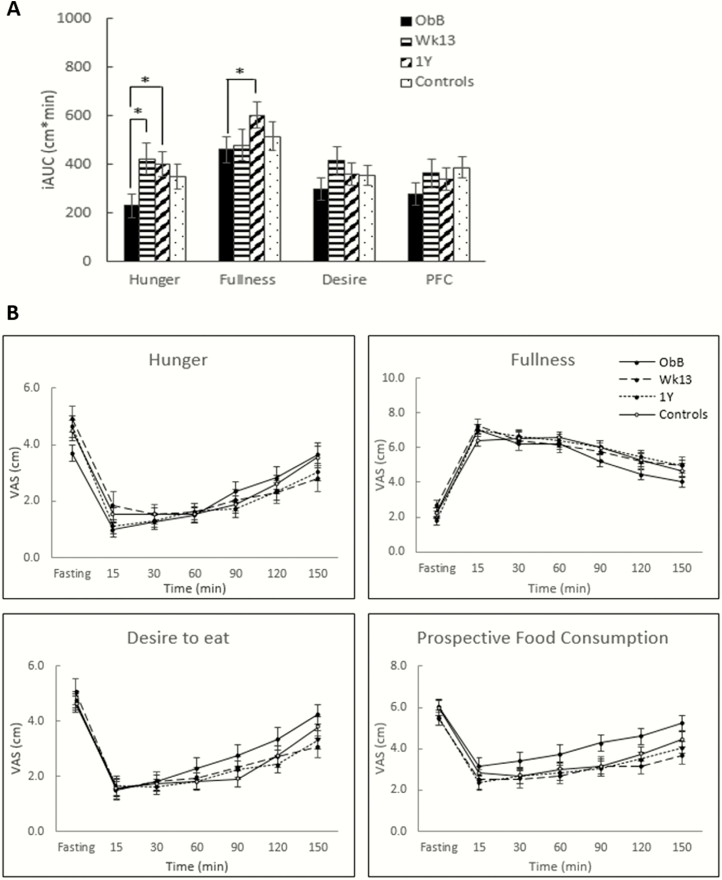
(A) Positive incremental AUC and (B) profiles over time for subjective feelings of appetite in the WL group at baseline (ObB), after 17% WL (week 13 and 1 year) and nonobese controls. AUC, area under the curve; PFC, prospective food consumption; WL, weight loss. Asterisks denote significant differences between groups: *P < 0.05, **P < 0.01, ***P < 0.001.

Conversely, postprandial feelings of hunger increased significantly with WL (both week 13 and 1 year) (*P* < 0.05 for both) compared with baseline, but these ratings were similar to that of the control group. Postprandial feelings of fullness were significantly greater after sustained WL (1 year) compared with baseline (*P* < 0.05), but no differences were seen between reduced-obese at 1 year and controls. No significant changes in subjective feelings of desire to eat or PFC in the postprandial state were seen over time in the obese group, nor significant differences between the obese group at any time point and controls (Fig. 1).

**Figure 2.**
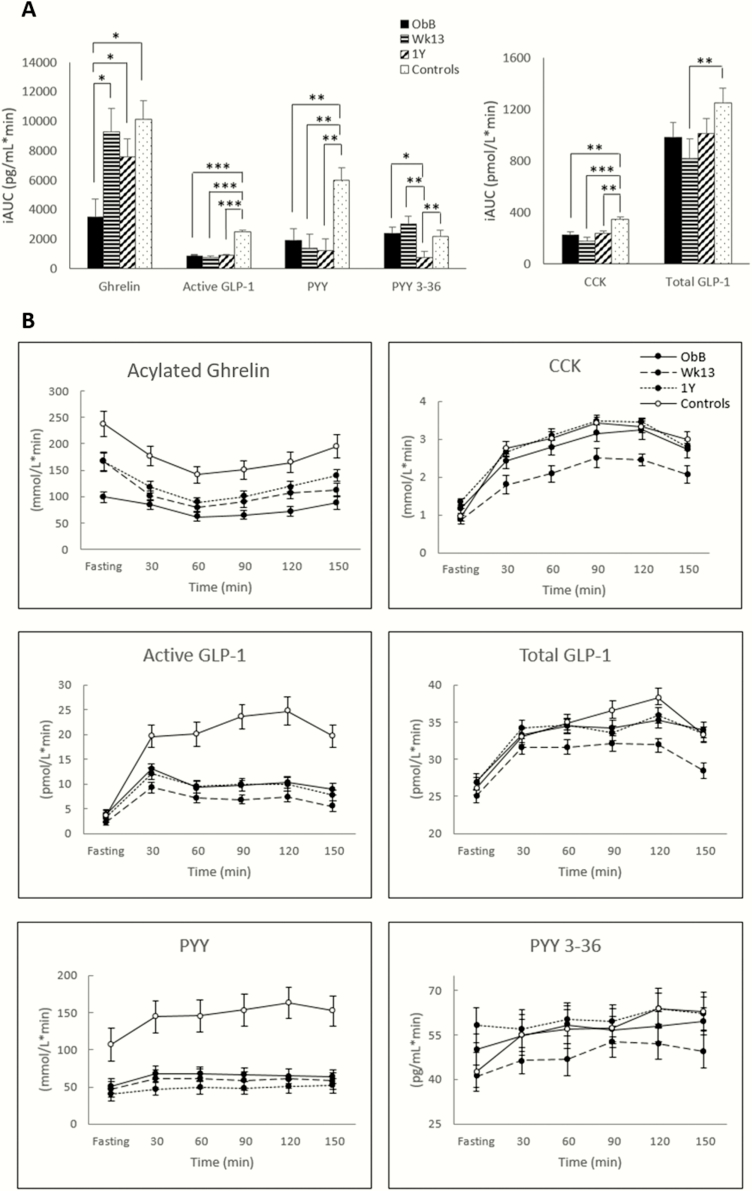
(A) Positive incremental AUC and (B) profiles over time for plasma concentrations of appetite-related hormones in the WL group at baseline (ObB), after 17% WL (week 13 and 1 year) and nonobese controls. AG, acylated ghrelin; AUC, area under the curve; CCK, cholecystokinin; GLP-1, glucagon-like peptide-1; PYY, peptide YY. Asterisks denote significant differences between groups *P < 0.05, **P < 0.01, ***P < 0.001.

The postprandial secretion of appetite-related hormones in obese, reduced-obese, and nonobese controls are presented in Fig. 2. Postprandial plasma concentration of ghrelin was also higher after WL (both week 13 and 1 year) compared with baseline (*P* < 0.05 for both), but to levels no different than those seen in nonobese controls. Individuals with obesity presented with significantly lower postprandial ghrelin secretion at baseline compared with nonobese controls (*P* < 0.05).

No significant changes in the postprandial secretion of active GLP-1, total PYY, or CCK were seen with WL over time. However, the control group presented with a significantly larger postprandial secretion of active GLP-1, total PYY, and CCK compared with both obese (baseline) and reduced-obese individuals (week 13 and 1 year) (*P* < 0.001 for active GLP-1 for both comparisons, *P* < 0.01 for total PYY for both comparisons and *P* < 0.001 and *P* < 0.01, respectively, for the comparison between obese at baseline and reduced-obese at week 13 and 1 year for CCK).

No significant changes were seen in the postprandial secretion of total GLP-1 with WL, but total GLP-1 iAUC was significantly lower in reduced-obese at Wk13 compared with controls (*P* < 0.01).

The postprandial secretion of PYY_3-36_ was significantly lower in reduced-obese at 1 year compared with both obese at baseline and reduced-obese at week 13 (*P* < 0.05 and *P* < 0.01, respectively). Moreover, the postprandial secretion of PYY_3-36_ was significantly lower in reduced-obese at 1 year compared with nonobese controls (*P* < 0.01)

## Discussion

This study aimed to test the hypothesis that the changes in homeostatic appetite markers concurrent with diet-induced WL are a normalization of hormone secretion toward what would be expected to be sensed at a lower body weight, FM, and FFM. To test this, we have compared subjective feelings of appetite and objective markers of appetite-related hormones in reduced-obese individuals with nonobese, FM, and FFM-matched controls.

We reported increases in postprandial hunger feelings and basal and postprandial ghrelin secretion in individuals with a sustained WL of 17%, but these were not different than those measured in controls. No significant increase in hunger feelings in the fasting state was seen in the present analysis. However, this is likely a result of lack of power, as previous research by our (12) and other research groups (6) in larger samples has shown that diet-induced WL leads to a sustained increase in hunger feelings in the fasting state. In general, the increased orexigenic response seen after WL in the present study, with both increased hunger feelings and secretion of ghrelin, has been previously reported by us and others (6, 10-12). These findings have served as the backbone of the compensation theory, suggesting that the increased orexigenic response seen with WL would lead to overeating and explain the recurring issue of weight recidivism. However, to our knowledge, this is the first paper to show that the increased orexigenic response seen in reduced-obese individuals is no different from and trends toward the levels seen in nonobese FM and FFM-matched controls, whereas hunger feelings at fasting appear blunted in obese at baseline.

In general, the postprandial secretion of satiety peptides did not change with WL, even though a reduction in PYY_3-36_ iAUC was seen with sustained WL (1 year) (both compared with baseline and week 13). Moreover, the postprandial secretion of satiety peptides was generally blunted in obese and reduced-obese (week 13 and 1 year) individuals compared with nonobese controls. The majority of the available evidence is consistent with our findings of a lower release of satiety peptides after a meal in individuals with obesity compared with nonobese controls (7, 18-21) even though some studies have reported no differences (22-25). Differences in BMI between groups and the hormonal fractions measured can potentially account for the majority of these inconsistencies between studies. Several studies (6, 10, 12, 26) have looked at the impact of diet-induced WL on the postprandial release of satiety peptides and the results are divergent, likely because of methodological differences related with the method of hormonal analyses and the specific fractions measured. For example, some found an increase in total GLP-1 (10, 11) and PYY_3-36_ (10) secretion in the postprandial state with WL, whereas others reported a decrease in total PYY and no changes in active GLP-1 (6, 12). Total PYY is a measure of secretion, whereas only PYY_3-36_ resulting from DPP-4 metabolism inhibits food intake (27). Similarly, total GLP-1 is the adequate measure of L-cell secretion, whereas intact GLP-1 only provides information about the “endocrine” part of the GLP-1 action, but not about the afferent signals (which total GLP-1 reflects) (28, 29).

Even though our results show overall a reduced postprandial secretion of satiety peptides in the reduced-obese compared with nonobese controls, which would go against the “normalization theory,” this occurred in the absence of significant differences in feelings of fullness after a meal between the 2 groups and our research group has, in fact, previously shown an increase in feelings of fullness in the postprandial state with sustained WL at 1 year follow-up, when compared with pre-WL values (12). Moreover, Verdich and colleagues (11), in what is, from our knowledge, the only previous study in which the concentration of appetite-related hormones in reduced-obese individuals after WL was compared with nonobese controls, reported a blunted total GLP-1 secretion in individuals with obesity versus healthy-weight controls, but no difference was seen between reduced-obese individuals (after WL) and controls. This suggests a normalization of GLP-1 postprandial secretion with WL.

A new theoretical approach to the biology of appetite control has recently been proposed by Blundell and colleagues (2015), where FFM and resting metabolic rate, in addition to signals arising from the adipose tissue and gut peptides play an important role in modulating food intake (30). This new approach suggests that FFM, as the main determinant of daily energy needs, constitutes a robust biological driver of food intake in individuals with obesity. Two major aspects make the translation of this new framework to our manuscript difficult. First, the main focus of our paper was to compare not individuals with obesity, but reduced-obese individuals with nonobese controls. Second, we measured appetite markers (both subjective feelings of appetite and plasma concentration of appetite-related hormones), not food intake. Food intake is very problematic to measure in free-living individuals. However, despite these caveats, our findings are in line with this new framework, as there were no differences in FFM (either kg or %) between reduced-obese and nonobese controls, which according to the new theoretical approach of the biology of appetite proposed by Blundell et al., would then mean no differences in the biological drive to eat between reduced-obese and nonobese FFM-matched controls.

This study has several strengths. First, the study design allowed for the evaluation of the impact of both recent (week 13) and sustained WL (1 year) in individuals with obesity on appetite markers, while also enabling for the comparison with a nonobese, FM- and FFM-matched control group. This is a unique design that not only contributes to an improved understanding of what changes in appetite are a result of physiological shifts because of recent WL and energy deprivation, and which changes reflect a newer stable and lower body weight. It needs to be emphasized that participants were in energy balance both at week 13 and 1 year, which is an extremely important asset when evaluating potential adaptations to the weight-reduced state. Moreover, this design allowed for the comparison between obese and nonobese weight-stable controls and also between reduced-obese and nonobese FM- and FFM-matched controls. As far as we are aware, this is the first study to measure different fractions of the same hormone (active and total GLP-1 and total PYY and PYY_3-36_), allowing for a broader overview of the impact of WL on the release of appetite-related hormones. Finally, the statistical analysis applied used strict cutoffs such as Bonferroni correction to restrict the likelihood of false-positive results.

Despite its strengths, this study has some limitations. Although specific assays have been used to measure CCK, total GLP-1, and PYY_3-36_, a multiplex kit was used to measure the other hormones, which is likely to result in less accurate and precise measurements than optimized assays for each individual hormone. In addition, the energy load of the standardized test meal was not adjusted for body size but was kept constant for all participants. This guarantees the same stimulus per participant, but does not account for the differing energy needs and body weight of each participant. Moreover, our manuscript focused only on episodic signals of appetite, and data on leptin, an important tonic signal, is absent, meaning that our view on appetite is unfortunately incomplete. We recognize that energy balance is a dynamic process and how energy is used is possibly different between those who have maintained a healthy body weight (controls) and those who are in a reduced obese state (31). Future studies could improve our understanding of metabolic adaptions to energy restriction by inclusion of a 4-compartment model of body composition, which would enable mathematical models of energy dynamics to be applied and further refined. Finally, even though our groups were matched for both FM and FFM, BMI was significantly lower in the control group.

The study has some important practical implications. We have previously shown that the changes in appetite that follow WL, in particular the increased hunger feelings and ghrelin secretion, are not predictors of weight regain in the long-term (9). These findings contradict the “compensation theory” (32-35), which suggests that the body fights against WL by upregulating ghrelin secretion and hunger feelings, which would then drive overeating and relapse in obesity management (weight regain). The results of the current study support our previous findings by showing that the increase in the orexigenic response following WL is in fact a “normalization” toward the expected levels at a lower body weight, FM, and FFM. Moreover, an improved satiety response has also been shown after WL, with increased release of satiety peptides postprandially (10), as well as increased feelings of fullness (12). This knowledge needs to be conveyed to health professionals, patients, and the general community. Individuals with obesity should expect to feel hungrier in the fasting state after they lose weight, but also more full after a meal. In summary, their appetite control system becomes more attuned to their lower body weight and sensing the reduction in fat mass. This likely represents a normalization toward what would be expected as fat mass reduces and, as we have previously shown, does not predict long-term relapse.

This concept needs, of course, to be tested and further validated because it represents a paradigm shift in thinking about relapse in obesity management, and that it is no longer an inevitable scenario resulting from increased orexigenic drive following diet-induced WL. Even though other biological pathways may be involved in weight recidivism, namely the hedonic appetite control system (36) or gut microbiota (37, 38), it is possible that it simply represents the struggle of reduced-obese individuals in adhering to a healthy lifestyle long-term, with reduced motivation and subsequent compliance with energy-restricted diets.

In conclusion, this analysis supports that the increase in the drive to eat (both hunger feelings and ghrelin secretion) observed with diet-induced WL is reflective of a normalization toward a lower body weight, FM and FFM, rather than a compensatory response.

## Abbreviations

AUC: area under the curve
BMI: body mass index
CCK: cholecystokinin
FFM: fat-free mass
FM: fat mass
GLP-1: glucagon-like peptide 1
iAUC: incremental area under the curve
PFC: prospective food consumption
PYY: peptide YY
VLED: very-low-energy diet
WL: weight loss

## Notes

**Study registration ID:** NCT01834859

**URL:** https://clinicaltrials.gov/ct2/show/NCT01834859

## Additional Information

***Disclosure Summary:*** The authors have no conflict of interest to disclose.
